# Does social trust stimulate university technology transfer? Evidence from China

**DOI:** 10.1371/journal.pone.0256551

**Published:** 2021-08-25

**Authors:** Ying Wu, Wen Huang, Li Deng

**Affiliations:** School of Finance, Southwestern University of Finance and Economics, Chengdu, China; University of Salento, ITALY

## Abstract

This paper examines the effect of social trust on university technology transfer. A large sample of Chinese universities from the 2007–2017 period was used. We find that social trust facilitates university technology transfer. The finding remain valid after a series of robustness. The mechanism test shows that social trust facilitates university technology transfer by improving the level of university-industry cooperative innovation. Our study suggests that social trust is an important factor that affects university technology transfer.

## Introduction

University technology transfer plays an important role in economic development [1–4]. However, the low level of university technology transfer is a serious issue Chinese universities face. According to the latest data of the Science and Technology Department of the Ministry of Education in China, in 2017, the investment in research and development of universities was nearly 154 billion yuan and the number of patents granted was 144375, but the number of patents sold to enterprises was 4803, which accounts for only approximately 3.3% of the total patents.

Given the importance of university technology transfer, a large body of literature investigates the factors that affect university technology transfer. However, the literature mainly concentrates on formal institutional factors, including university technology transfer offices [5, 6], industry-university cooperative research centers [7, 8], incubators [9, 10], and the law [11, 12]. Previous studies also show that informal institutions also affect university technology transfer, such as the academic culture [13] and geographic reach [14]. Social trust, as one important informal institution, affects economic and social development [15–21]. Nevertheless, we know little about the impact of social trust on university technology transfer. To fill this research gap, this study investigates the role of social trust in university technology transfer. Specifically, we examine the association between social trust and university technology transfer.

Trust is a person’s subjective assessment of the probability that certain actions will be conducted by another party. Culture is an important factor that affects social trust [22]. Fifty-six ethnic groups within thirty-one provinces and more than eighty different native dialects make the level of social trust have a large regional variation in China [18], which provides the opportunity to examine the impact of social trust on university technology transfer. There are a number of potential reasons why social trust can affect university technology transfer.

One reason is related to the reduction of the transaction costs of technology transfer. The university technology transfer process can result in transaction costs, including material costs, information costs, and negotiation costs [23]. Besides, the demand side of technologies might have a larger tendency to engage in opportunism to acquire technology without payment in the process of understanding technical details, which hinders technology transfer and results in transaction costs. Social trust makes negotiation more successful in the technology market [24] and constrains opportunistic behavior [25], thus reducing the transaction costs of technology transfer. In summation, social trust can prompt university technology transfer by reducing transaction costs.

Another reason is related to university-industry cooperative innovation. A lower ability to independently innovate and bear risk makes university industry cooperative innovation an important channel for boosting university technology. Higher levels of social trust are associated with more cooperation among collaborators [26, 27]. Cooperative innovation between universities and industries allows technologies to meet the market demand and reduce demand-side seeking, which is conducive to university technology transfer. Based on the above analysis, we expect social trust to stimulate university technology transfer through university-industry cooperative innovation.

Following the previous literature on social trust in China, we use two social trust proxies in this paper. The first proxy, Trust, comes from a survey by the Chinese Enterprise Survey System Across China in 2000, which measures the perceived provincial trustworthiness of companies located in thirty-one provinces in China [16–18, 21, 28, 29]. The second proxy, Trust1, is the city-level trustworthiness in 2017, taken from the China Urban Commercial Credit Environment Index; this is calculated by the China Academy of Management Science [30]. We use Trust as the main measure of social trust in our empirical tests and Trust1 as an alternative measure in the robustness tests. Regarding the dependent variable, we use the natural logarithm of the number of patent transfer contracts to measure university technology transfer.

We use the data of technology transfer from 770 Chinese universities for the 2007–2017 period to test the impact of social trust on university technology transfer. We document extensive evidence that social trust is positively related to university technology transfer. The evidence is robust to controlling for region attributes and changing measures of social trust and university technology transfer. Universities located in a region with higher levels of social trust have more technology transfers than those that are not. Moreover, we only test whether social trust affects university technology through the channel of university-industry cooperative innovation because we cannot obtain data on the transaction costs in technology transfer. We find that social trust stimulates university technology transfer through university-industry cooperative innovation.

There is only one paper that is similar to ours. Jensen et al. examine the impact of trust on the market for technology [24]. They use survey responses from university-firm and firm-firm technology transactions and find that high levels of trust are more likely to conclude a transaction. However, our paper differs from this study in two aspects. First, we use the data of Chinese universities’ technology transfer to test the impact of social trust on university technology transfer instead of Australian data. Second, we highlight and test the channel through which social trust affects university technology transfer, which is not included in the paper of Jensen et al. [24].

This article contributes to the existing literature in several ways. First, we empirically test whether and how social trust affects university technology transfer in China. To the best of our limited knowledge, no previous paper has examined the impact of social trust on university technology transfer in China. The paper fills the gap. Second, we expand the work of Jensen et al. [24], which examines the effect of trust on university technology transfer in Australia. We use Chinese data to study the impact of trust on university technology transfer and find that social trust affects university technology transfer by prompting university-industry cooperative innovation. Third, we enrich the research on the impact of informal institutions on university technology transfer. The previous literature find that academic culture [13] and geographic reach [14] can affect university technology transfer. We examine how informal institutions affect university technology from the perspective of trust. Finally, we expand the research of social trust. The literature on the impact of social trust mainly focuses on economic growth [31, 32] and corporate finance [16–18, 21, 33]. Our paper expands the scope of the impact of social trust.

The remaining sections of the paper are organized as follows. Section 2 is the background, literature review, and hypothesis. Section 3 introduces the sample, variables, and empirical methodology. Section 4 presents the empirical results. Section 5 reports the results of the channel test. Section 6 concludes.

## Background, literature review, and hypothesis

### University technology in China

China built modern western-style universities in the 1890s. It was not until 1930 that scientific and engineering training was set up in Chinese university system [34]. Before the establishment of the People’s Republic of China in 1949, teaching was primarily task in universities. From the 1949 to early 1980s, university technology transfer was controlled by government. Government provided R&D projects and decided which enterprises received universities’ innovation output. Technologies were not merchandise in the marketplace [35]. Since the economic reform in 1979, China has been moving from central planned economy to market socialism. Universities obtained greater autonomy in managing their R&D projects. In 1987, China passed the Technology contract Law and the government’s right to license major scientific and technological outputs in universities was abolished. In 1996, the enactment of law of Promoting the Transformation of Scientific and Technological Achievements was milestone for Chinese universities’ technology transfer, which encouraged universities to engage more commercialization of technological outputs. To further promote university technology transfer, government issued the Regulations on Promoting the Transformation of Scientific and Technological Achievements in 1996, improving the reward proportion for inventors and reducing tax for technology transfer. In 2001, the Ministry of Education and the State Economic and Trade Commission jointly established state technology transfer centers in six universities. The next milestone in Chinese university technology transfer was in 2015. Government revised the law of Promoting the Transformation of Scientific and Technological Achievements, which expanded the universities’ power of research results obtained with government funding and increasing the reward standard from 20% to 50% for inventors in technology transfer.

After decades of development, China has established a complete university technology transfer system. In addition to general transfer organization, such as university technology transfer offices, incubators, industry-university cooperation research centers, and science parks, China developed an additional form, namely, university-affiliated enterprises [36]. Although nearly all Chinese universities have technology transfer organizations, the rate of technology transfer is always lower than western countries.

### Related literature on trust

The literature on the impact of social trust mainly focuses on economic growth and corporate finance. We will review the studies of trust in the following two aspects.

Using data on generalized trust obtained from the World Survey database, Zak and Knack find that social trust can promote economic growth by boosting investment [37]. Bjornskov states that social trust can affect economic growth through schooling and the rule of law [31]. Horvath uses Bayesian model averaging and data on nearly fifty countries to examine the impact of social trust on economic growth and finds that trust has a positive effect on long-term growth [15]. Lv et al use an interaction model with data from 27 industries in 31 Chinese provinces from the 2000 to 2010 period to test whether social trust affects economic growth and find that social trust positively affects economic growth [32].

Wu et al find that private firms located in regions with higher social trust use more trade credit from suppliers, collect receivables and pay payables quicker, and provide more trade credit to customers [16]. Using data on Chinese listed firms for the 2001–2015 period, Li et al examine the impact of trust on stock price crash risk and find that firms headquartered in regions with high social trust have smaller crash risk because managers have more honest behaviors (e.g., higher accounting conservatism and fewer financial restatements) [18]. Meng and Yin use firm data from 22 countries to test the effect of trust on debt costs and find that firms in countries with a higher level of social trust tend to have lower bond yield spreads [33]. Kong et al point out that trust has a positive effect on corporate innovation [21].

In summary, the literature has paid little attention to university technology transfer. This paper extends the social trust literature and analyses the role social trust plays in university technology transfer.

### Related literature on university technology transfer

Regarding the impact of formal institutions on university technology transfer, Markman et al show that the ability of university technology transfer offices to commercialize technology in the U.S. is positively associated with the scale of licensing revenue streams [5]. However, Swamidass and Vulasa find that university technology transfer offices might slow the rate of commercialization of university inventions due to the lack of adequately trained staff and patent processing capacity in university technology transfer offices [38]. In terms of industry-university cooperative innovation, Adam et al find that industry-university cooperation research centers can promote technology transfer between universities and firms [7]. Villani et al use an Italian case study to examine the impact of university incubators on university technology transfer and find that university incubators can promote university technology transfer by reducing the social and geographical distance between universities and industries [10]. Link and Hasselt examine the impact of the Bay-Dole Act of 1980 on university technology and show that the law affects the internal transfer of technology from universities by providing universities with an incentive to invest in technology transfer offices [12].

Regarding the impact of informal institutions on university technology transfer, Feldman and Desrochers suggest that we should consider the academic culture of universities when we study the role of universities in technology transfer because the academic culture of a university can affect the technology transfer process [13]. Friedman and Silberman find that universities located in regions with many large and growing technical firms have a higher rate of technology transfer [14]. Boh et al note that graduate and postdoctoral students also play important roles in university technology transfer [39]. Using data from university-firm and firm-firm technology transactions, Jensen et al find that trust can improve technology transfer [24].

### Hypothesis

University technology transfer is a type of transaction process. Transaction costs are general in university technology transfer [23]. Transaction costs are based on three assumptions: bound rationality, opportunism and asset specificity [40]. Bound rationality means that individuals cannot forecast perfectly and draw up complete contracts. Asset specificity means that investments can create positive returns. Social trust makes universities provide enterprises with more detailed and accurate information about patents, which helps enterprises reduce the uncertainty of future operations and assess the return of patents. Besides, social trust also makes individuals’ behavior more moral [18] and constrains opportunism [25]. The reduction of opportunism alleviates the concern of universities that enterprises acquire technology without payment. In summation, social trust can improve the levels of university technology transfer by reducing transaction costs.

Limited by various conditions, many enterprises have a low ability to perform independent innovation and take risks, which makes university-industry cooperative innovation an important way to accelerate university technology transfer. Universities and enterprises have different understandings of the market. Universities usually pay more attention to academic research instead of the demand market, which makes it difficult for universities to find buyers for patents. University-industry cooperative innovation can allow the technologies produced by universities to meet the market demand and reduce the technology seeking of enterprises. Thus, university-industry cooperative innovation facilitates technology transfer [7]. In addition, a higher level of social trust is associated with more cooperation among collaborators [26]. Successful university-industry collaboration innovation requires trust [41]. In summation, social trust can facilitate university technology transfer by improving the levels of university-industry cooperative innovation.

Based on the above analysis, social trust can promote university technology transfer by reducing transaction costs and improving the levels of university-industry cooperative innovation. We propose the following hypothesis.

#### Hypothesis

Social trust stimulates university technology transfer.

## Sample and empirical methodology

### Sample

We obtain information on university technology transfer from the Report on the Statistics of Science and Technology in Universities, which is compiled by the Department of Science and Technology, Chinese Ministry of Education. The report presents detailed data on the technology activities of all universities. The sample period is 2007–2017 because the Ministry of Education began issued detail information of university R&D activities in 2007 and stopped in 2017. and Then, we exclude university-year observations with missing information for control variables and merge the data of universities that changed names. Our final sample includes 7035 university-year observations representing 770 universities. We also winsorize the continuous variables at the 1% and 99% levels to reduce the impact of outliers. Following prior studies [16–18, 21], we use the first proxy of social trust (Trust) obtained from the survey in 2000 by Zhang and Ke [42]. Following Liu et al. [30], we use the proxy of social trust (Trust1) obtained from the survey in 2017 by the Chinese Academy of Management Science in a robustness test. Other data come from the Chinese Research Data Services (CNRDS) database.

### The measure of university technology transfer

Because patent licensing is the main method of technology transfer, we use the natural logarithm of the number of university technology contracts plus one to measure the levels of university technology transfer (Transfer) in the main regression due to the left skewness of patent licensing. In the robustness test, we use the natural logarithm of university technology transfer incomes plus 1 to measure the levels of university technology transfer (Transfer1).

### The measure of social trust

We define a dummy variable to measure social trust in the main regression. Specifically, we regard the provinces that obtained scores in the top 10 in the “Chinese Enterprise Survey System” survey in 2000 by Zhang and Ke [42] as regions with higher social trust (Trust). Then, if the provinces are in the top 10, the variable Trust1 equals 1; otherwise, it equals 0. The questionnaires were delivered to over 15000 enterprise managers located in the 31 provinces, and more than 45000 useful responses were obtained. The main question of trust is the following: “Based on your experience, Could you list order the top five provinces where the enterprises are most trustworthy?”. The raw score is 5 for the top ranking, 4 for the second ranking, and so on. The trust score for each province is the weighted average trustworthiness ranking given by managers. For example, Shanghai is ranked the top by 22.7% of the responding managers, second by 16.5%, third by 8.7%, fourth by 6%, and fifth by 3.7%. Then, the trust score of Shanghai is calculated as (22.7%×5+16.5%×4+3×8.7%+2×4.8%+1×3.7% = 218.9%).

In the robustness test, following Liu et al. [30], we use the commercial credit environment index of the capital of each province in the survey of the “China urban commercial credit environment index” in 2017 by the Chinese Academy of Management Science to measure social trust (Trust1) because credit can largely reflect the level of trust. The index covers seven dimensions: credit investment, the enterprise credit management function, the credit reference system, government credit supervision, breach of credit behavior, honesty education and enterprise feelings. The index ranges from 0 to 100. The larger the value is, the better the city’s business trust environment is.

### Empirical model

To test the impact of social trust on university technology transfer, we examine the following model.

$${Transfer}_{i,t}=\alpha_{0}+\alpha_{1}{Trust}_{i,t}+\gamma ’{Control}_{i,t}+\mu_{i}+v_{t}+\varepsilon_{i,t}$$ (1)

where i represents the year and t represents university. The dependent variable, Transfer_i,t_, is measured by Transfer in the main test and by Transfer1 in the robustness test. The control variables (Control) are potential factors that might affect university technology transfer. We control the type of university (Type), size of university (Size), research and development staff (Resp), technology achievements (Product), the number of enterprises(Industry), regional economic development (GDP), and government policy (Law). Moreover, we also include regional fixed effects (μ_i_) to control unobservable time-invariant region-specific characteristics and year-fixed effects (υ_t_) to control common time trends. All of these variables are defined in S1 Appendix.

## Empirical test and results

### Summary statistics

Table 1 represents the summary statistics of the variables used in our regression. The table shows that the mean and maximum values of the variable Transfer are 1.472 and 5.481, respectively, which is in accordance with the fact that some universities, such as Tinghua University, have higher R&D investment and technology transfer abilities. Trust has a mean value of 0.439, suggesting that only 43.9% of the samples come from regions with higher levels of social trust. The mean and maximum values of Product are 6.230 and 9.251, respectively, which show that there are large differences of research abilities in universities. In summary, the results of the statistical description show that the characteristics of Chinese universities’ scientific research activities are heterogeneous.

**Table 1:** Summary statistics.

Variable	N	Mean	Std	Min	Median	Max
Transfer	7035	1.091	1.472	0.000	0.000	5.481
Trust	7035	0.439	0.496	0.000	0.000	1.000
Type	7035	0.161	0.368	0.000	0.000	1.000
Size	7035	6.495	1.045	3.664	6.485	9.150
Resp	7035	5.395	1.391	1.386	5.537	8.499
Product	7035	6.230	1.320	2.303	6.280	9.251
Industry	7035	9.265	1.019	6.155	9.428	11.070
Law	7035	0.308	0.462	0.000	0.000	1.000
GDP	7035	10.591	0.527	9.396	10.596	11.680

The table reports the result summary statistics of the variables. The sample period is 2007–2017, and the sample size is 7035. All variables are defined in S1 Appendix.

### Pearson correlation matrix

Table 2 reports the Pearson correlation matrix of the variables. As shown in Table 2, the correlation coefficient between social trust and university technology transfer is 0.145, which is positive and statistically significant at the 1% level. This preliminary finding shows that social trust is conducive to university technology transfer. In addition, the correlation coefficient between social trust and university technology transfer is not high, indicating that multicollinearity is not a serious problem.

**Table 2:** Pearson correlation matrix.

	Transfer	Trust	Type	Size	Resp	Product	Industry	Law	AGDP
Transfer	1								
Trust	0.145***	1							
Type	0.394***	0.088***	1						
Size	0.481***	0.150***	0.435***	1					
Resp	0.506***	0.202***	0.449***	0.866***	1				
Product	0.554***	0.153***	0.497***	0.880***	0.830***	1			
Industry	0.182***	0.544***	-0.084***	0.120***	0.105***	0.114***	1		
Law	0.019	-0.007	-0.028	-0.060***	-0.045***	-0.090***	0.012	1	
GDP	0.187***	0.527***	0.137***	0.124***	0.189***	0.130***	0.319***	0.411***	1

The table represents the Pearson correlation matrix. *, **, and *** indicates statistical significance at the 10%, 5%,1% level,respectively. The sample period is 2007–2017, and the sample size is 7035. All variables are defined in S1 Appendix.

### Main regression

Table 3 reports the estimates of the main regressions using Eq (1). Column 1 reports the results of the regression in which we do not control for other variables. The coefficient of Trust is positive and statistically significant at the 1% level, which shows that social trust facilitates university technology transfer. In column 2, we control the type of university (Type), the size of the university (Size), research and development staff (Resp), and technology achievements (Product). The coefficient of Trust is still positive and statistically significant at the 1% level. In column 3, we further control for the number of enterprises(Industry), regional economic development (GDP), and government policy (Law). The coefficient of Trust1 is also positive and statistically significant at the 1% level, which supports our hypothesis that Trust facilitates university technology transfer.

**Table 3:** The impact of social trust on university technology transfer.

	(1)	(2)	(3)
	Transfer	Transfer	Transfer
Trust	1.057***	0.365***	0.410***
	(7.903)	(3.637)	(2.891)
Type		0.656***	0.656***
		(12.516)	(12.523)
Size		-0.212***	-0.214***
		(-6.612)	(-6.658)
Resp		0.215***	0.216***
		(10.902)	(10.918)
Product		0.462***	0.462***
		(18.323)	(18.317)
Industry			-0.167
			(-1.626)
Law			0.177
			(0.776)
GDP			0.251
			(1.193)
Constant	0.753***	-1.953***	-2.776*
	(7.777)	(-15.719)	(-1.821)
Province fixed effects	Yes	Yes	Yes
Year fixed effects	Yes	Yes	Yes
N	7035	7035	7035
R^2^	0.113	0.390	0.390

The table reports the results of the main regressions. The sample period is 2007–2017, and the sample size is 7035. The t-statistics are presented in parentheses. The symbols *, **, and *** indicate statistical significance at the 10%, 5% and 1% levels, respectively. All variables are defined in S1 Appendix.

In terms of the control variables, there is no evidence that the size of a university facilitates university technology transfer, which might be because Chinese universities blindly pursue becoming comprehensive universities by expanding the size of the university but do not pay more attention to improving their technology transfer ability. Besides, the regression coefficient of Product is positive and statistically significant at the 1% level, indicating that stronger research ability of universities has a positive effect on technology transfer. Finally, we fail to find that the “Law of the People’s Republic of China on the Promotion of Application of Scientific and Technological Achievements” in 2015 can facilitate university technology transfer.

### Robustness tests

In this subsection, we run three robustness tests. First, in column 1, we use Transfer1 to measure university technology transfer. Specifically, we use the natural logarithm of university technology transfer income plus 1 to measure the levels of university technology transfer. The coefficient of Trust is positive and statistically significant at the 1% level, which is consistent with our hypothesis.

In column 2, we use Trust1 to measure social trust. Specifically, we use the natural logarithm of the China urban commercial credit environment index in 2017 conducted by the Chinese Academy of Management Science. The coefficient of Trust1 is positive and statistically significant at the 1% level, which is consistent with our hypothesis.

The impact of social trust on university technology transfer might be due to important variables being missing. Enterprises’ ability to absorb technology is an important factor that might affect university technology transfer. We use the R&D expenditure of large enterprises divided by total assets in a province where universities are located to measure the absorptive technology of enterprises (Absorptive). We control the variable in Eq (1) and the results of the regression are shown in column 3 of Table 4, suggesting that social trust significantly promotes university technology transfer.

**Table 4:** Robustness tests.

	(1)	(2)	(3)
	Transfer1	Transfer	Transfer
Trust	1.421***		0.416***
	(4.092)		(2.676)
Type	1.398***	0.656***	0.683***
	(11.743)	(12.523)	(12.074)
Size	-0.647***	-0.214***	-0.211***
	(-7.703)	(-6.658)	(-6.046)
Resp	0.688***	0.216***	0.213***
	(13.315)	(10.918)	(9.878)
Product	1.228***	0.462***	0.456***
	(19.310)	(18.317)	(16.719)
Industry	-0.326	-0.167	-0.145
	(-1.258)	(-1.626)	(-1.170)
Law	0.608	0.177	0.151
	(1.049)	(0.776)	(0.625)
GDP	0.279	0.251	0.256
	(0.528)	(1.193)	(1.034)
Trust1		2.420***	
		(2.891)	
Absorptive			0.0290
			(0.535)
Constant	-4.820	-13.131***	-3.014*
	(-1.273)	(-4.384)	(-1.689)
Province fixed effects	Yes	Yes	Yes
Year fixed effects	Yes	Yes	Yes
N	7035	7035	5869
R^2^	0.415	0.390	0.401

The table reports the result of robustness tests. Column 1 reports the regression results after replacing the dependent variable. Column 2 reports the results after replacing the independent variables. In column 3, we consider the effects of important variables being missing. The reduction of sample size in column 3 is due to some data on R&D expenditure of large enterprises in the region where universities are located being missing. The sample period is 2007–2017. The t-statistics are presented in parentheses. The symbols *, **, and *** indicate statistical significance at the 10%, 5% and 1% levels, respectively. All variables are defined in S1 Appendix.

## Mechanism test

In the previous portions of the paper, we show that social trust can promote university technology transfer by reducing transaction costs and improving the level of university-industry cooperative innovation. We can only test whether social trust facilitates university technology transfer by improving the level of university-industry cooperative innovation because transaction costs cannot be measured using existing data. We use the amount of funding supported by enterprises and institutions divided by the total amount of funding that universities obtain to measure university-industry cooperative innovation (Cooperation).

Following Ye et al. [43], we use the following method to examine the underlying mechanism. First, we use Eq (2) to test whether social trust affects the level of university-industry cooperative innovation. We expect the coefficient of Cooperation to be positive and significant. Then, we add the variable Cooperation into Eq (3). We expect a reduction in the coefficient of Trust or a less significant of the coefficient of Trust1.

$${Cooperation}_{i,t}=\beta_{0}+\beta_{1}{Trust}_{i,t}+\gamma \prime {Control}_{i,t}+\mu_{i}+\nu_{t}+\varepsilon_{i,t}$$ (2)

$$\begin{array}{c} {Transfer}_{i,t}=\lambda_{0}+\lambda_{1}{Trust}_{i,t}+\lambda_{2}{Cooperation}_{i,t}\\ +\gamma^{'}{Control}_{i,t}+\mu_{i}+\nu_{t}+\varepsilon_{i,t} \end{array}$$ (3)

where we control the variables that might affect university-industry cooperative innovation in Eq (2). Specifically, we control the type of university (Type), the size of the university (Size), research and development staff (Resp), and technological achievements (Product). In Eq (3), all control variables are the same as those in Eq (1).

As shown in Table 5, in column 1, we find that the coefficient of Trust is positive and statistically significant at the 1% level, which shows that social trust improves the level of university-industry cooperative innovation. In column 2, the coefficient of Trust is positive and statistically significant at the 5% level. Furthermore, the coefficient of Trust in column 2 is smaller and less significant than that in column 3 of Table 3, which shows that social trust can facilitate university technology transfer by improving the level of university-industry cooperative innovation. Moreover, the coefficient of Cooperation is positive and statistically significant at the 1% level, which is consistent with Adam et al. [7].

**Table 5:** Mechanism tests.

	(1)	(2)
	Cooperation	Transfer
Trust	0.099***	0.288**
	(5.209)	(2.129)
Type	-0.030***	0.697***
	(-4.184)	(13.800)
Size	-0.015**	-0.193***
	(-2.546)	(-6.458)
Resp	0.013***	0.199***
	(3.210)	(10.285)
Product	0.047***	0.399***
	(10.352)	(16.528)
Cooperation		1.352***
		(17.154)
Industry		-0.185*
		(-1.846)
Law		0.197
		(0.894)
GDP		0.264
		(1.297)
Constant	-0.093***	-2.623*
	(-4.345)	(-1.772)
Province fixed effects	Yes	Yes
Year fixed effects	Yes	Yes
N	7035	7035
R^2^	0.193	0.420

The table represents the results of the mechanism test. The sample period is 2007–2017, and the sample size is 7035. The t-statistics are presented in parentheses. The symbols *, **, and *** indicate statistical significance at the 10%, 5% and 1% levels, respectively. All variables are defined in S1 Appendix.

## Conclusion

This paper examines whether social trust affects university technology transfer. Using a sample of Chinese universities over the 2007–2017 period, we find that social trust facilitates university technology transfer. After a series of robustness tests, including using alternative measures of social trust and technology transfer and further controlling the factors that might affect university technology transfer, the results of our paper are still valid. Moreover, our empirical result supports that social trust can facilitate university technology by improving the level of university-industry cooperative innovation.

Our paper has important implications for the literature focusing on university technology transfer. Previous studies have mainly focused on the impacts of formal institutions on university technology transfer. This paper shows that social trust, an important informal institution, can stimulate university technology transfer. Scholars can pay more attention to other informal institutions that might transfer university technology.

## Supporting information

S1 Appendix Variable definitions. (DOCX)

S1 Data (ZIP)
