# Supplementary material for: Does social trust stimulate university technology transfer? Evidence from China
Source: PLoS One. 2021 Aug 25;16(8):e0256551. doi: 10.1371/journal.pone.0256551 (PMC8386828; doi:10.1371/journal.pone.0256551)
Supplement: S1 Appendix — (DOCX) [file pone.0256551.s001.docx]

S1 Appendix: Variable definitions

| Variable |  |
| --- | --- |
| Transfer | The natural logarithm of the number of university technology contracts plus 1 |
| Transfer1 | The natural logarithm of university technology transfer incomes plus 1 |
| Trust | A dummy variable that equals 1 if province obtained a score in top 10 in the 2000 survey by Zhang and Ke(2002) and otherwise 0 |
| Trust1 | The natural logarithm of the score of the capital of the province in the 2017 survey by Chinese Academy of Management Science |
| Type | A dummy variable that equals 1 if a university belongs to “211” project and 0 otherwise. |
| Size | The natural logarithm of the number of university staff members |
| Resp | The natural logarithm of the number of research and development staff members |
| Product | The natural logarithm the number of papers of a university |
| Industry | The natural logarithm of the number of enterprises |
| Law | A dummy variable that equals 1 after 2015 and 0 otherwise. |
| GDP | The natural logarithm of GDP in a province |
| Absorptive | R&D expenditure divided by total assets in large industries enterprises |
| Cooperation | The amount of funding supported by enterprises and institutions divided by total the amount of funding that a university obtains |
